# Electrospun polycaprolactone nanofibrous membranes loaded with baicalin for antibacterial wound dressing

**DOI:** 10.1038/s41598-022-13141-0

**Published:** 2022-06-28

**Authors:** Weiwei Zeng, Nga-man Cheng, Xia Liang, Haofeng Hu, Fulin Luo, Jia Jin, Ya-wei Li

**Affiliations:** 1 The Second People’s Hospital of Longgang District, Shenzhen, 518112 China; 2 Shenzhen Baoan Women’s and Children’s Hospital, Jinan University, Shenzhen, 518102 China; 3 Accident and Emergency Medicine Academic Unit, The Chinese University of Hong Kong, Hong Kong, SAR China; 4 College of Life Sciences and Medicine, Zhejiang Sci-Tech University, Hangzhou, 310018 China; 5 Lianshui People’s Hospital, Affiliated to Kangda College of Nanjing Medical University, Huaian, 223400 China

**Keywords:** Biomaterials, Health care

## Abstract

Due to the rise in bacterial resistance, the antibacterial extractions from Chinese herbs have been used more frequently for wound care. In this work, baicalin, an extraction from the Chinese herb *Scutellaria baicalensis*, was utilized as the antibacterial component in the poly(*ε*-caprolactone)/MXene (PCL/Ti_3_C_2_T_X_) hybrid nanofibrous membranes for wound dressing. The results revealed that the presence of Ti_3_C_2_T_X_ aided in the diameter reduction of the electrospun nanofibers. The PCL hybrid membrane containing 3 wt% Ti_3_C_2_T_X_ nanoflakes and 5 wt% baicalin exhibited the smallest mean diameter of 210 nm. Meanwhile, the antibacterial tests demonstrated that the PCL ternary hybrid nanofibers containing Ti_3_C_2_T_X_ and baicalin exhibited adequate antibacterial activity against the Gram-positive bacterial *S. aureus* due to the good synergistic effects of Ti_3_C_2_T_X_ naoflakes and baicalin. The addition of Ti_3_C_2_T_X_ nanoflakes and baicalin could significantly improve the hydrophilicity of the membranes, resulting in the release of baicalin from the nanofibers. In addition, the cytotoxicity of the nanofibers on rat skeletal myoblast L6 cells confirmed their good compatibility with these PCL-based nanofibrous membrances. This work offers a feasible way to prepare antibacterial nanofibrous membranes using Chinese herb extraction for wound dressing applications.

## Introduction

The skin is the first barrier for the human body to protect the internal organs against microorganisms or other external hazards^1^. However, the skin is highly susceptible to damage due to injury or illness. Cutaneous wounds are easily infected, resulting in a massive burden on the healthcare system^2^. Traditional wound dressing materials such as cellulose, silk, alginate, collagen, and so forth, have no ability to inhibit bacteria colonization or avoid microorganisms’ growth^3–5^. Therefore, there is a need for antibacterial wound dressings to prevent cutaneous wound contamination. Recently, electrospun nanofiber scaffolds have attracted considerable attention in the field of wound dressings due to their unique characteristics, such as high oxygen permittivity, high tensile strength, diverse morphological features, tunable porosity and tailored ability^6^. Antibacterial ingredients such as antibiotics, metal oxides, and active carbon nanoparticles are being incorporated into the fibrous matrix to aid in the healing of cutaneous wounds^7,8^.

Poly(*ε*-caprolactone) (PCL) is a type of biodegradable and biocompatible aliphatic linear polyester that can be synthesized by the ring-opening polymerization of ε-caprolactone. PCL has received considerable attention due to its high toughness, biodegradability, and biocompatibility. It has been demonstrated that electrospun PCL nanofibrous scaffolds can be utilized for wound dressing applications^9–11^. The native porous structure characteristics of PCL scaffolds can mimic the skin's extracellular matrix (ECM) structural properties while also providing high oxygen permeability. In order to confer antibacterial capabilities on the PCL nanofibrous scaffolds, different types of metal oxides or metals have been introduced into the PCL matrices^12^. Zhu et al. found that the insertion of silver (Ag) and magnesium (Mg) ions into gelatin/polycaprolactone (GT/PCL) could endow the nanofibers with antibacterial activity as well as pro-angiogenesis function, which benefited for skin wound repair^13^. Ghiyasi and his colleagues discovered that the hybrid scaffolds consisting of Urtica dioica, ZnO nanoparticles and PCL had good antibacterial activity against *E. coli* and *S. aureus*^14^. In addition, the nanofiber of the hybrid scaffold exhibited good biocompatibility and cell adhesion to fibroblast L929 cells in vivo tests. Ekram et al. demonstrated that the presence of zinc chloride (ZnCl_2_) reduced the diameter of the PCL/ZnCl_2_ nanofibers while increasing the degradation rate and mechanical properties^15^. Moreover, the antibacterial composite nanofiber was found to greatly boost the proliferation of stem cells. Trcin et al. found that the PCL scaffolds containing TiO_2_ nanoparticles could provide statistically significant antimicrobial activity against different types of bacteria^16^. Furthermore, the PCL/TiO_2_ scaffolds with a maximum porosity of 93%, on the other hand, were found to be capable of supporting the adhesion and proliferation of limbal stem cells.

MXene is a new family of two-dimensional (2D) materials that integrates the transition metals M (Ti, Cr, V, Nb and Mo etc.) with huge amounts of X (carbides, nitrides, or carbonitrides) by removing the A-element in the MAX phase^17,18^. MXene (Ti_3_C_2_T_X_) exhibits superior biocompatibility and antibacterial efficiency against both Gram-negative and Gram-positive bacteria than graphene oxide due to its ultrathin structure and unique physiochemical properties^19,20^. Awasthi et al. found that the addition of MXene into PCL nanofibers maintained good biocompatibility in vitro with ibroblasts (NIH-3T3) and preosteoblasts (MC3T3-E1) cell lines^21^. In addition, the presence of Ti_3_C_2_T_X_ nanosheets contributed to reducing the diameter and improving the morphology of PCL/Ti_3_C_2_T_X_ nanofibers. However, no data on the antibacterial activities of PCL/Ti_3_C_2_T_X_ nanofibers have been reported to far.

Herbal extracts have been widely utilized to cure various diseases since ancient times. Plant-derived phytochemicals can serve as potential antibacides with fewer side effects. Baicalin, a flavonoid extracted from the Chinese herb Scutellaria baicalensis, has been regarded as a multitherapeutic agent in the field of biomedicine^22^. It shows various positive benefits on wounds, including anti-oxidative, anti-bacterial, and anti-inflammatory properties^23,24^. However, little research has been performed to date on the use of baicalin in electrospun fibers for wound dressings.

In this work, MXene (Ti_3_C_2_T_X_) was first exfoliated to obtain Ti_3_C_2_T_X_ nanoflakes. Then the resulting Ti_3_C_2_T_X_ nanoflakes were incorporated into the PCL matrix with herbal extraction baicalin by electrospinning. The morphology, thermal stability, hydrophilicity, and mechanical properties of the electrospun nanofibers consisting of PCL/Ti_3_C_2_T_X_/baicalin ternary composites were investigated. The addition of Ti_3_C_2_T_X_ and baicalin was expected to have synergistic effects on improving the wound dressing's antibacterial performance against gram-positive bacterial *S*. *aureus*. Furthermore, the biocompatibility in vitro of this wound dressing was also evaluated by using rat skeletal myoblast L6 cells.

## Materials and methods

### Materials

Poly(ε-caprolactone) (PCL, CapaTM 6800) with a mean molecular weight of 80,000 was obtained from Weibo Chemical Co., Ltd. (Guangzhou, China). MXene (Ti_3_C_2_T_X_) with 400 meshes was supplied by Beike 2D materials Co., Ltd. (Beijing, China). Baicalin (purity > 95%) was supplied by Macklin Biochemical Co., Ltd. (Shanghai, China). Chloroform and dimethylformamide (DMF) were purchased from J&K (Beijing, China).

### Preparation of electrospun PCL composite nanofibrous membranes

The exfoliation of Ti_3_C_2_T_X_ was performed by high energy ball milling using a Miqi YXQM-1L planetary micromill (Changsha, China). Ti_3_C_2_T_X_ (4 g) and zirconium oxide milling balls (70 g) were placed in a 250 ml grinding bowl and performed at 600 rpm for 4 h. Then the exfoliated Ti_3_C_2_T_X_ was removed from the grinding bowl with DI water, followed by sonication in DI water for 30 min to exfoliate completely. Finally, the resulting Ti_3_C_2_T_X_ nanoflakes were centrifuged at 3500 rpm for 10 min, and the obtained suspension was freeze-dried overnight.

**Figure 1:**
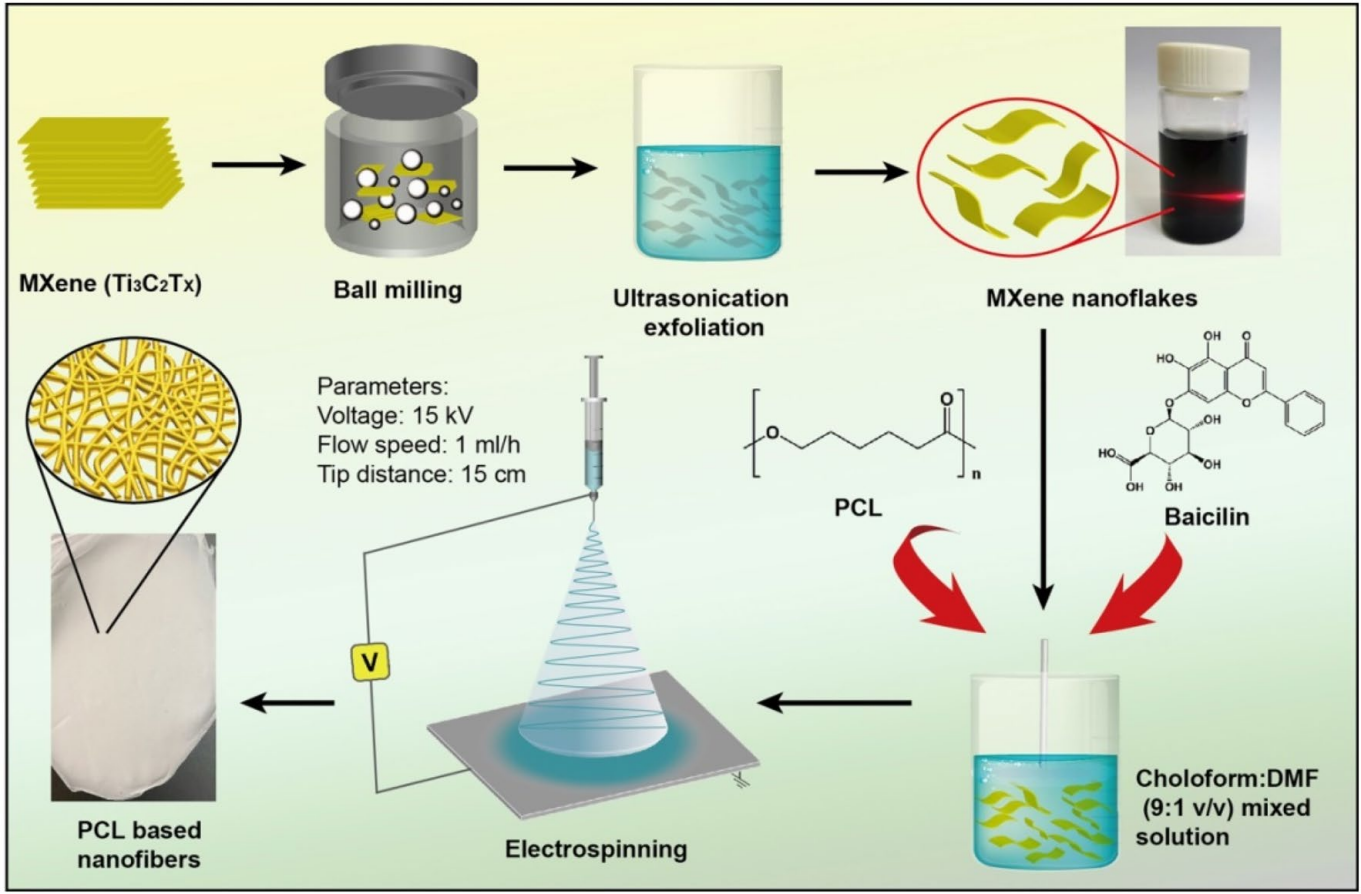
Preparation route of PCL based nanofibers. (Adobe Illustrator CS5, Version 15.0.0, https://www.adobe.com/products/illustrator.html).

The electrospun nanofibers of the PCL/Ti_3_C_2_T_X_/baicalin ternary composite were prepared using a commercially available electrospinning machine (TL-Pro, Tongli Weina Co., Ltd., Shenzhen, China), as shown in Fig. 1. The formulations of PCL based nanofibers are shown in Table 1. The concentration of PCL was fixed at 100 mg/mL in a mixed chloroform/DMF (8:2, v/v) solution. Subsequently, the desired amount of Ti_3_C_2_T_X_ and baicalin was added into the PCL solution with vigorous stirring. The prepared PCL solution was loaded into a 10 mL plastic syringe with a metal capillary needle (0.50 mm inner diameter, and 30 mm length). The applied electrospinning voltage was fixed at 15 kV and the flow rate was kept at 1 mL/h. The obtained nanofibers were subsequently placed in a vacuum oven at 50 ℃ for 6 h to remove the remaining solvent.

**Table 1:** Formulations of PCL based nanofibers.

Samples	PCL (wt%)	Ti_3_C_2_T_X_ (wt%)	Baicalin (wt%)
PCL-0	100	–	–
PCL-1	97	3	–
PCL-2	97	–	3
PCL-3	94	3	3
PCL-4	92	3	5

### Scanning electron microscope (SEM) observation

The morphology of Ti_3_C_2_T_X_ and the prepared nanofibers was observed by a scanning electron microscope (SEM, FEI Quata 250, USA). Prior to observation, the specimens were sputtered with a thin layer of gold to avoid charge accumulation.

### Atomic force microscope (AFM) observation

The thickness of Ti_3_C_2_T_X_ nanoflakes was measured by an atomic force microscopy (AFM, VEECO Multimode V, USA) with tapping mode.

### X-ray diffraction (XRD) analysis

The X-ray diffraction patterns were conducted on a grazing-incident XRD (Rigaku SmartLab) with Cu *K*_α_ at 45 kV. The scanned angle (2*θ*) ranged from 5° to 60°.

### Fourier-transform infrared (FT-IR) analysis

The Fourier-transform infrared (FT-IR) spectra were performed with a Perkin Elmer FTIR-100 spectrometer (USA) with a collected wavenumber range of 500–4000 cm^−1^.

### Thermogravimetric analysis (TGA)

The thermal stability was evaluated by a thermogravimetric analyzer (TGA, Netzsch TGA-209F1). The specimens were heated from room temperature to 600 ℃ at a ramping rate of 10 ℃/min.

### Water contact angels (WCA)

The wettability of the nanofibers was evaluated via the measurement of the water contact angle using a See System E instrument (Advex Instruments, Czech Republic).

### Minimum inhibitory concentration (MIC) determination

The standard broth microdilution method was applied to determine the MIC value of baicalin toward *S. aureus* as described in the previous reports^25,26^. Briefly, the *S. aureus* was inoculated and grown in a broth subculture inside a flask. The bacteria were then incubated at 37 °C for 24 h. Then, the bacterial concentration was adjusted to a density of 1.0 × 10^6^ CFU/ml. The baicalin solution, with a concentration ranging from 1 to 1024 mg/ml, was added into the *S. aureus* solution to observe the bacteria’s growth. The lowest concentration, at which no visible bacterial growth was observed in the plate, was considered as the MIC.

### Antibacterial tests

The antibacterial properties of the PCL based nanofiebers against *S. aureus* were evaluated by a standard “SNV 195920-1992” evaluation model^26^. The evaluation standard of inhibition zone is summarized in Table S1. The Firstly, 100 µL of 10^8^ CFU/mL bacteria suspension was spread on an LB agar plate, and then the nanofibrous membrane samples with a diameter of 1.0 cm were placed on the surface of the agar. The bacteria suspension with the PCL-based films were incubated at 37 °C, followed by the digital images of the PCL-based nanofibrous membranes on the agar plate with bacteria were recorded at 24 h, 72 h, and 120 h, respectively. All samples were tested in triplicate. 

### Cytocompatibility evaluation

The cytotoxicity of the nanofibers on rat skeletal myoblast L6 cells was evaluated using the CCK-8 method with a leaching pattern. The sterilized nanofibers extract solutions (10 mg/mL) were prepared by immersing the dried nanofiber in medium for 12 h at 37 ℃ with ultrasonic extraction. The L6 cells were seeded in a 96-well plate at a density of 5000 cells/well, and pre-cultured for 24 h before replacing the culture medium with the fresh medium and extract solutions to make the final sample concentration of 0.2 mg/mL, 1 mg/mL, and 5 mg/mL. Each sample to be tested (PCL-0, PCL-1, PCL-2, PCL-3, and PCL-4), blank control (culture medium) and positive control (culture medium and cell) were incubated for 48 h and repeated four times. The results were recorded as the absorbance at 450 nm through an ultraviolet spectrophotometer by the following formula:

$${\text{Cell Growth Rate }}\left( {{\text{RGR}}} \right)\, = \,\left( {{\text{Test}}_{{{\text{OD45}}0}} -{\text{ Blank}}_{{{\text{OD45}}0}} } \right)/\left( {{\text{Positive}}_{{{\text{OD45}}0}} -{\text{ Blank}}_{{{\text{OD45}}0}} } \right)\, \times \,{1}00\% .$$

### Statistical analysis

All experiments were carried out in triplicate. The data was analyzed by the SPSS software (IBM Analytics, USA). Significance of all the statistical tests was predetermined at P < 0.05. Results were expressed as mean ± standard deviation (SD).

## Results and discussion

### Characterization of MXene nanoflakes

**Figure 2:**
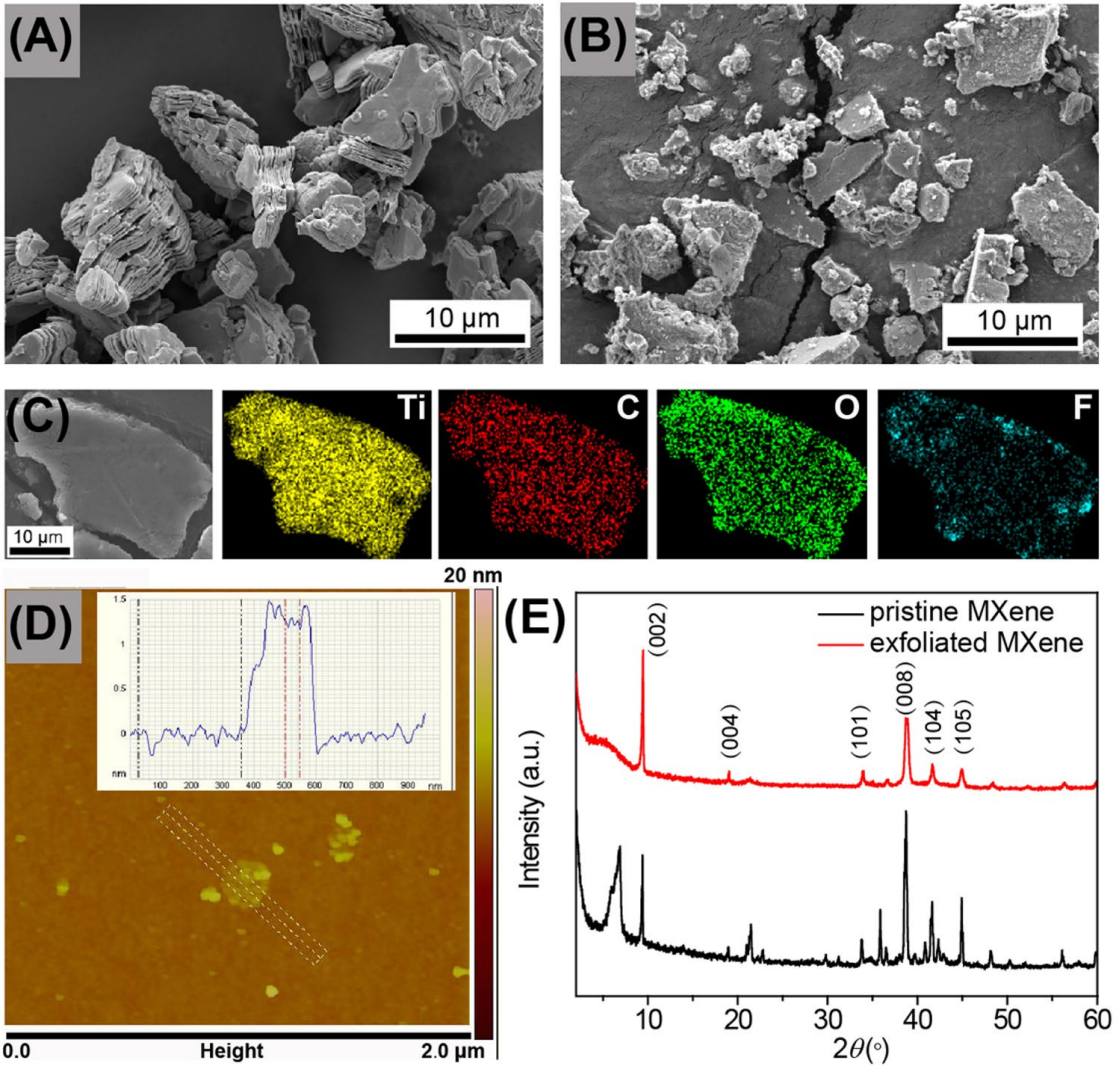
SEM images of **(A)** pristine Ti_3_C_2_T_X_, **(B)** exfoliated Ti_3_C_2_T_X_ nanoflakes; **(C)** EDX elemental mapping of exfoliated Ti_3_C_2_T_X_ nanoflakes, **(D)** AFM image of exfoliated Ti_3_C_2_T_X_ nanoflakes, and **(E)** XRD patterns of pristine Ti_3_C_2_T_X_ and exfoliated Ti_3_C_2_T_X_ nanoflakes.

In Fig. 2A, the pristine MXene (Ti_3_C_2_T_X_) shows a typical organ-like structure with gaps of tens of nanometers wide. The exfoliated Ti_3_C_2_T_X_ nanoflakes in Fig. 2B show that the layers are clearly separated from each other. In addition, some small nanoflakes in the range of tens of nanometers are observed due to the grinding. The EDX elemental mapping images in Fig. 2C confirm the presence of Ti, C, and O, which is consistent with the chemical structure of Ti_3_C_2_T_X_^20^. The appearance of F on the surface of Ti_3_C_2_T_X_ nanoflakes is due to the remaining F element after etching with LiF/HCl^17^. In the AFM image in Fig. 2D, it is observed that the exfoliated Ti_3_C_2_T_X_ nanoflake has a thickness of 1.5 nm, which is similar to the reported results^27,28^. The XRD patterns of pristine Ti_3_C_2_T_X_ and exfoliated Ti_3_C_2_T_X_ nanoflakes are shown in Fig. 2E. It is noted that the characteristic peak at 6.9° in pristine MXene indicates the interlayer spacing of 1.28 nm. In addition, the prominent peaks at 9.4°, 19.1°, 34.0°, 38.7°, 41.7° and 44.9°, which correspond to the diffraction of (002), (004), (101), (008), (104), and (105) planes of Ti_3_C_2_T_X_, respectively^29^. As for the exfoliated Ti_3_C_2_T_X_ nanoflakes, the characteristic peak shifts from 6.9° to 5.4° due to the exfoliation. In addition, this peak becomes board and weak, which is ascribed to the ball milling and exfoliation reducing the size of the nanoflakes and enlarging the interlayer spacing.

### Characterization of PCL nanofibrous membranes

**Figure 3:**
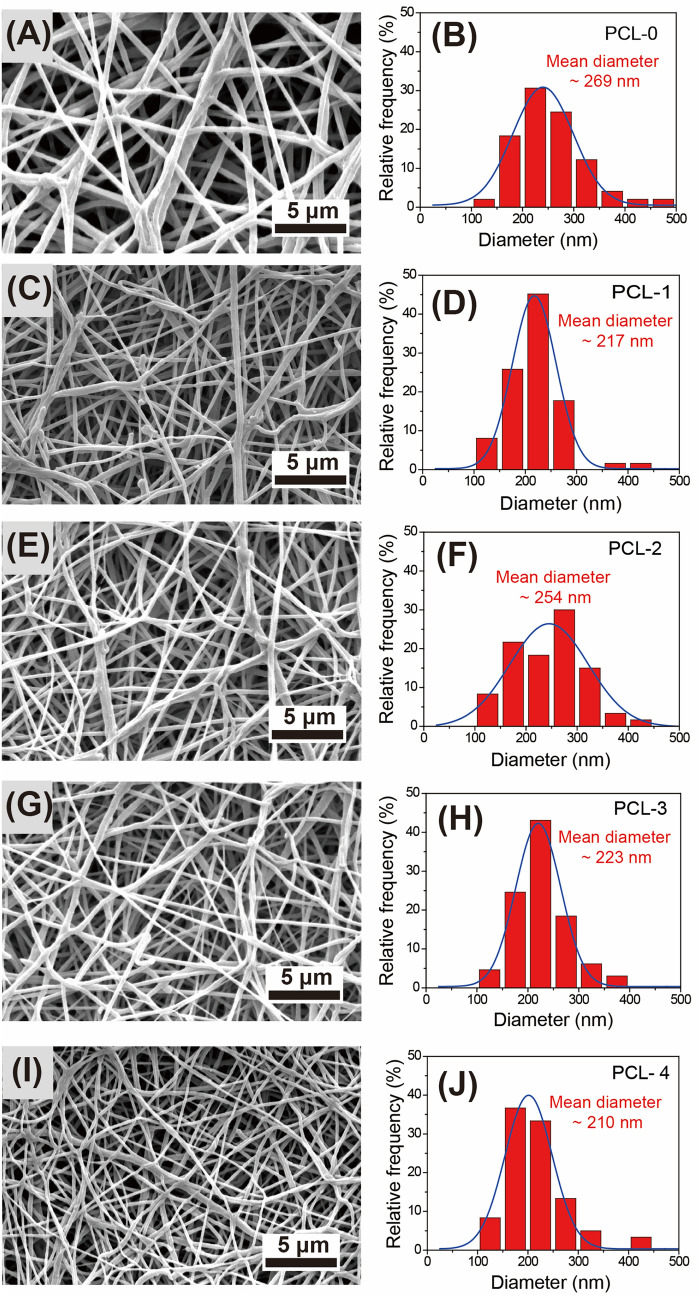
SEM images of electrospun PCL based nanofibrous membranes and measured fiber diameter distribution: **(A,B)** PCL-0, **(C,D)** PCL-1, **(E,F)** PCL-2, **(G,H)** PCL-3, and **(I,J)** PCL-4.

The SEM images of PCL nanofibrous membranes are presented in Fig. 3. It is noted that the nanofibers of PCL-0 with a mean diameter of 269 nm (Fig. 3B) have a smoother surface and non-beaded structures. With the addition of 3 wt% Ti_3_C_2_T_X_ nanoflakes, the mean diameter of PCL-1 decreases from 269 to 217 nm as compared with that of PLA-0. In Fig. 3F,H,J, the mean diameters of PCL-2, PCL-3 and PCL-4 are 254 nm, 223 nm, and 210 nm, respectively. It is believed that the presence of conductive Ti_3_C_2_T_X_ nanoflakes can increase the charge density of the electrospinning solution due to the polar groups on their surface, thus enhancing the electrostatic force of the applied electric field and reducing the diameter of the nanofibers^30,31^. On the other hand, the introduction of baicalin has little effect on the diameter of the nanofibers. However, the surface of the PCL nanofibers containing baicalin is relatively smoother than that of PCL-1. It is speculated that baiclin is a type of small molecule with many hydroxyl groups that can reduce the viscosity of the electrospinning solution and react with the polar groups of Ti_3_C_2_T_X_ nanoflakes to form hydrogen bonds, resulting in the homogeneous dispersion of Ti_3_C_2_T_X_ nanoflakes in the PCL matrix.

The typical tensile stress versus strain curves for PCL-based nanofibrous membranes are shown in Fig. S1A, and the corresponding tensile properties are summarized in Fig. S1B. It is noted that the pure PCL membrane shows high ductility (elongation at break of 305%), which is consistent with other published works^32,33^. For PCL-1, the addition of Ti_3_C_2_T_X_ nanoflakes results in strong reinforcing effects, increasing the tensile modulus significantly. Although the abundant polar groups on the surface of Ti_3_C_2_T_X_ nanoflakes can react with the ester bonds of PCL, the inhibition effects of the rigid nanosheets will be more profound, lowering the plastic flow ability of PCL molecular chains^34^. On the other hand, the PCL-2 shows a clear decrease in both elasticity and strength. In Figure S1B, the elongation at break and tensile strength of PCL-2 are reduced to 178% and 2.58 MPa, respectively. This is because baicalin is a small molecule that has a plasticizing effect on PCL. As for PCL-3, the tensile strength of the samples improved obviously, with a little sacrifice in elongation at break as compared with those of PCL-2. It is speculated that the presence of Ti_3_C_2_T_X_ nanoflakes can react with baicalin to form hydrogen bonds to some extent. With the further increase in the baicalin content, the tensile properties of PCL-4 deteriorate progressively, indicating the baicalin exceeds the reaction sites of Ti_3_C_2_T_X_ nanoflakes.

**Figure 4:**
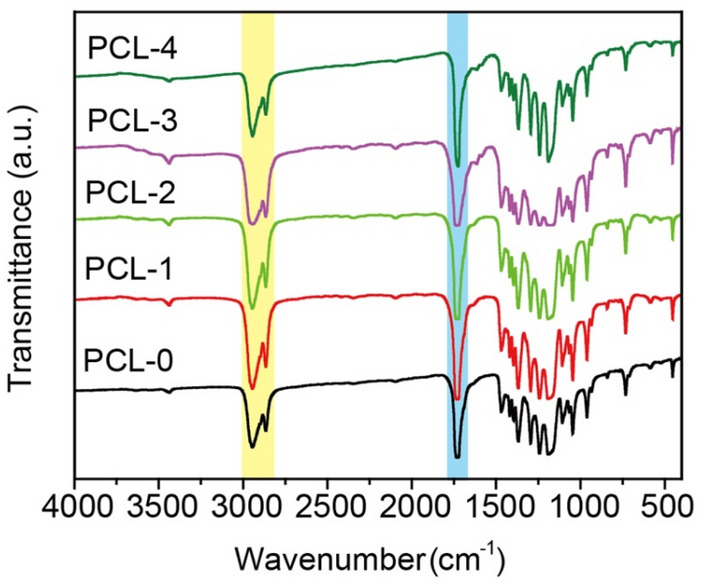
FT-IR spectra of PCL-based nanofibrous membranes.

The FT-IR spectra of PCL-based nanofibrous membranes are presented in Fig. 4. It is clearly observed that the two characteristic peaks located at 2943 cm^−1^ and 2863 cm^−1^ of PCL-0 correspond to the stretching bands of CH_2_ groups. The absorption peak at 1737 cm^−1^ is ascribed to the stretching of the C=O groups, while the peaks at 1294 cm^−1^ and 1184 cm^−1^ belong to the asymmetric and symmetric stretching of the C–O–C groups, respectively. In addition, the characteristic peak at 1243 cm^−1^ denotes the CH_3_ vibrations, and 1045 cm^−1^ belongs to C–O stretching and C–H bending^35^. With the incorporation of Ti_3_C_2_T_X_ and baicalin, no obvious change is observed in the FT-IR spectra. The ternary composites show a weak and broad peak between 3600 and 3300 cm^−1^, which is ascribed to the overlapping of the O–H polar groups on the surface of Ti_3_C_2_T_X_ and the O–H stretching vibration of baicalin^36^. Furthermore, there is a new characteristic peak at 1609 cm^−1^ that corresponds to the stretching vibration of C=C of phenyl groups from baicalin^37^.

**Figure 5:**
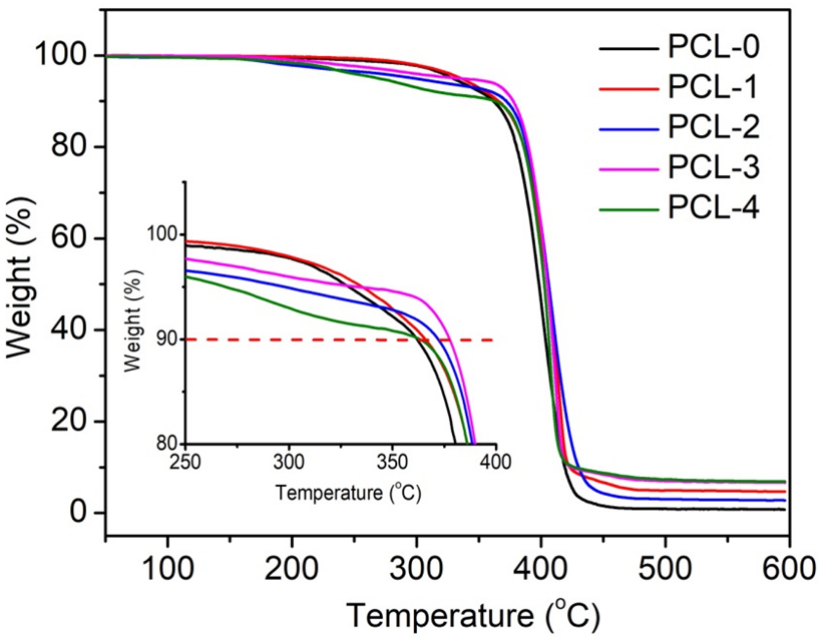
TGA curves of PCL-based nanofibrous membranes.

The thermal stability of PCL-based nanofibrous membranes was measured by TGA, as shown in Fig. 5. The corresponding data, including the initial weight loss temperature (*T*_10_, the temperature at 10% weight loss), the peak weight loss temperature (*T*_p_, the temperature at maximum weight loss rate), and char residues at 600 ℃ are listed in Table 2. It can be observed that PLA-0 shows a distinct weight loss stage from 300 to 420 ℃, which was associated with the pyrolysis of the chemical bond cleavage of PCL chains^38^. The thermal decomposition curve of PCL-1 shifts to a higher temperature, indicating the presence of Ti_3_C_2_T_X_ can increase the thermal stability of PCL nanofibers. According to Table 2, the *T*_10_ and *T*_p_ of PCL-1 increases from 361.8 to 365.8 ℃ and from 403.2 to 415.7 ℃, respectively, as compared with that of PLA-0. This is due to the Ti_3_C_2_T_X_ nanoflakes serving as a thermal barrier to protect the underlying PCL matrix. In the case of the PCL/Ti_3_C_2_T_X_/baicalin ternary nanofibers, the thermal decomposition curves can be roughly divided into two major stages. The initial weight loss stage occurs in the range of 200–370 ℃ due to the thermal decomposition of baicalin. The second stage occurs at around 370–420 ℃, which is related to the pyrolysis of PCL chains. In addition, the *T*_10_ and *T*_p_ of PCL-4 show a decreasing tendency as compared with those of PLA-0, which is ascribed to the low thermal stability of baicalin.

**Table 2:** TGA data of PCL-based nanofibrous membranes.

Samples	*T*_10_ (℃)	*T*_p_ (℃)	Residues at 600 ℃ (wt%)
PCL-0	361.8	403.2	0.73
PCL-1	365.8	415.7	4.71
PCL-2	372.1	408.5	2.79
PCL-3	377.7	412.2	6.65
PCL-4	363.2	407.9	7.38

**Figure 6:**
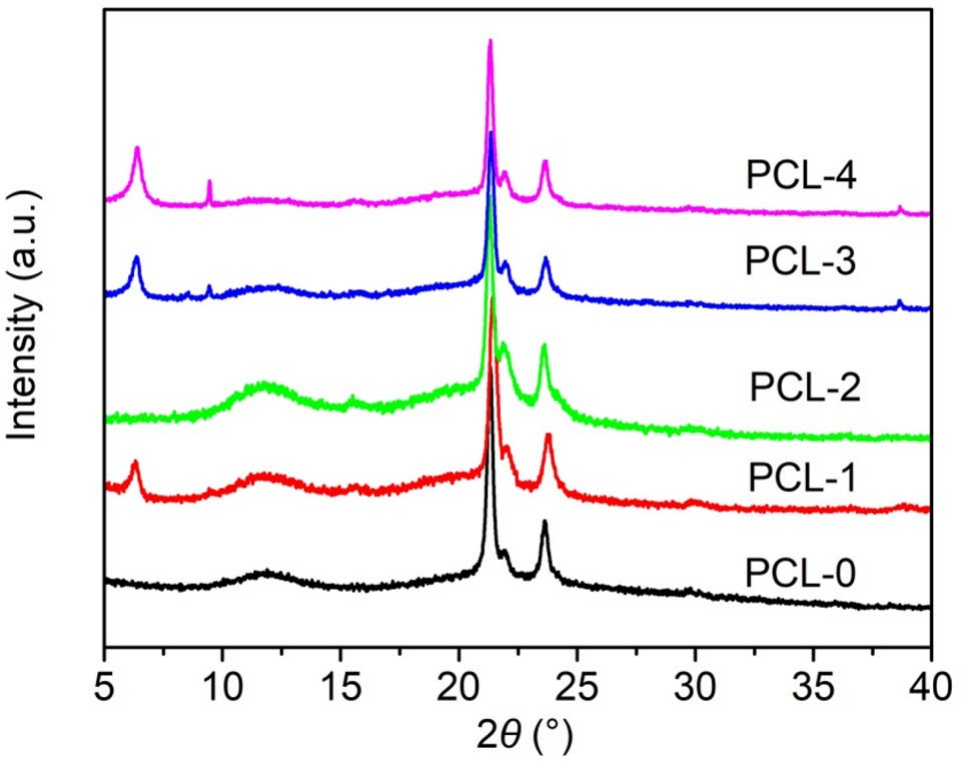
XRD patterns of PCL-based nanofibrous membranes.

Figure 6 depicts the XRD patterns of PCL-based nanofibrous membranes. As shown in Fig. 6, the PCL-0 has three significant diffraction peaks at 2*θ* = 21.3°, 22.0° and 23.7°, which correspond to the (110), (111), and (200) planes, respectively^39^. In addition, there is also a relatively weak and broad peak at 11.8° in the PCL-0 pattern. The above results indicate that the pristine PCL is in a semi-crystalline state. In the XRD pattern of PCL-1, one additional peak appears at 2*θ* = 6.4°, which is attributed to the (002) plane of Ti_3_C_2_T_X_^20^. After the introduction of baicalin, the diffraction angle of PCL at 2*θ* = 11.8° becomes weaker and shifts to a lower angle, indicating that the physical interaction between PCL and baicilin is altered.

**Figure 7:**
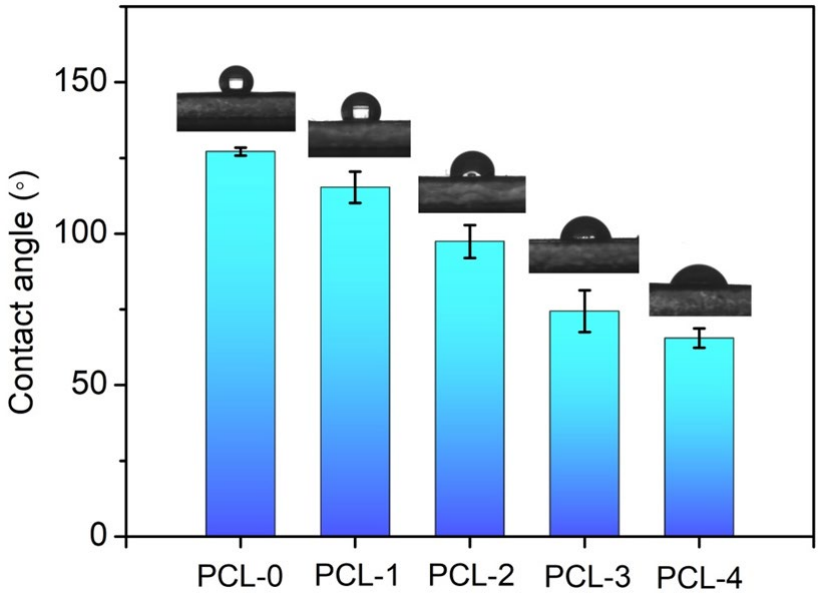
Water contact angle of PCL-based nanofibrous membranes.

The water contact angles (WCA) of the membrane surfaces were measured to evaluate the hydrophilicity of the PCL-based nanofibrous membranes, as shown in Fig. 7. It is observed that PCL-0 has a hydrophobic surface with a WCA of 127.1° ± 1.3°, which is consistent with previous reports^40,41^. With the addition of Ti_3_C_2_T_X_ nanoflakes, the WCA of PCL-1 shows a slight decrease (115.3° ± 5.2°) due to the abundance of −OH groups on the surface of *Ti*_*3*_*C*_*2*_*T*_*X*_. When baicilin was introduced into the PCL matrix, the surfaces of the PCL nanofibrous membrances shifted to hydrophobicity. The contact angles for the PCL-2, PCL-3, and PCL-4 are 82.4° ± 5.4°, 74.4° ± 6.9°, and 65.5° ± 3.2°, respectively. It can be attributed to the fact that baicalin contains a large number of hydrogel groups that increase the hydrophilicity of the electrospun fibers.

### Antibacterial activities of PCL nanofibrous membranes

**Figure 8:**
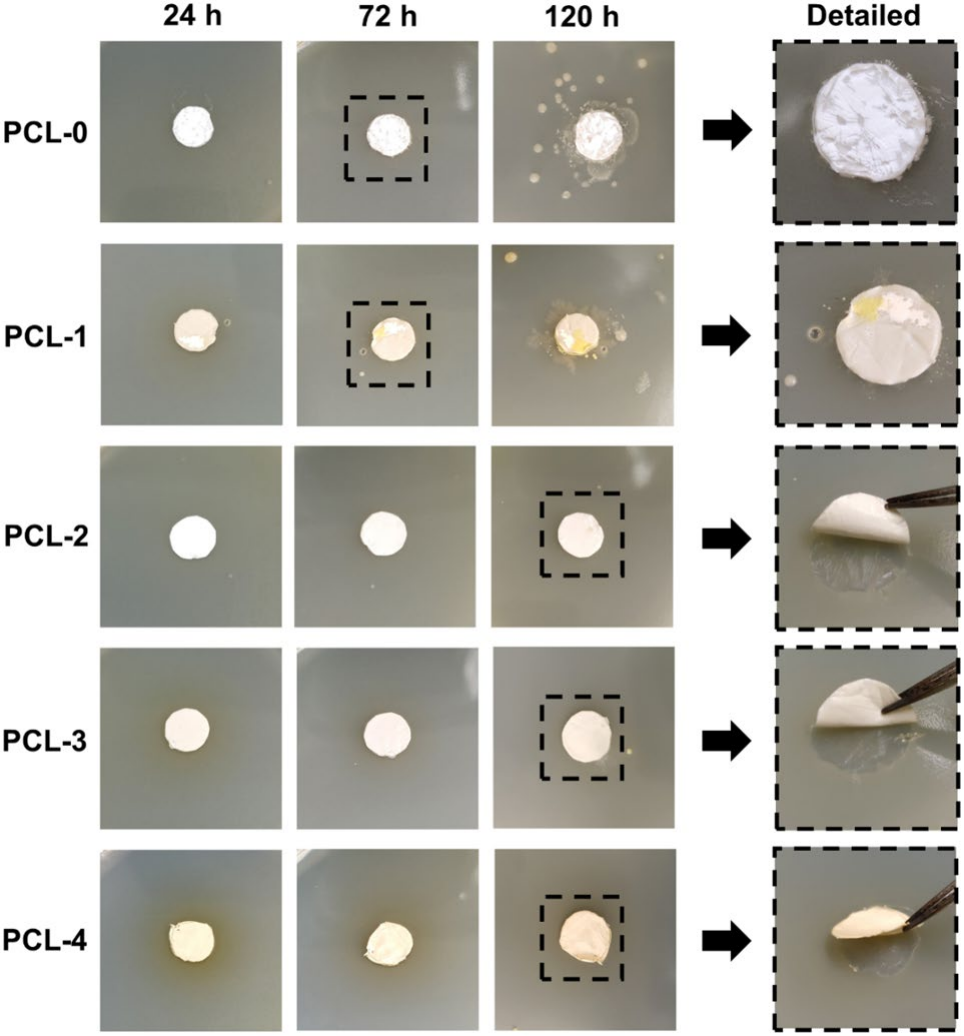
Antibacterial performance of PCL-based membranes against *S. aureus*. (Microsoft Office, PowerPoint 2010, https://www.microsoft.com/zh-hk/microsoft-365/previous-versions/office-2010).

Baicalin is the main antibacterial constituent of PCL-based membranes, which has broad-spectrum antibacterial activity, especially against Gram-positive bacteria like *S. aureus*^42^. In this work, the MIC of baicalin against *S. aureus* was found to be effective at 32 mg/ml, which is similar to the reported literatures^43,44^. All PCL-based nanofibrous membranes were selected for a standard antibacterial test by the preincubation plate pouring method. In Fig. 8, the bacterial colonies appear clustered around the PCL-0 and PCL-1 nanofibrous membranes after 72 h in the detailed pictures, and more remarkable clustered colonies are observed after 120 h. This is due to PCL having no antibacterial ability, which is consistent with previous report^45^. On the other hand, PCL-2, PCL-3, and PCL-4 show obvious bacteriostatic activity at 120 h incubation time (The medium dried completely after 120 h, and further inhibited bacterial growth). It can be ascribed to the presence of baicalin can interrupt the formation of α-heptamer, hindering the cell lysis activity of *α*-Hemolysin of *S. aureus*^46^. In addition, it is observed that the surrounding areas of PCL-3 and PCL-4 exhibit a light yellow color at 24 h, indicating the addition of Ti_3_C_2_T_X_ nanoflakes contributed to the spread of baicalin from the nanofibers. That is because baicalin has poor solubility in aqueous solution^47^.

### Biocompatbility of PCL nanofibrous membranes

**Figure 9:**
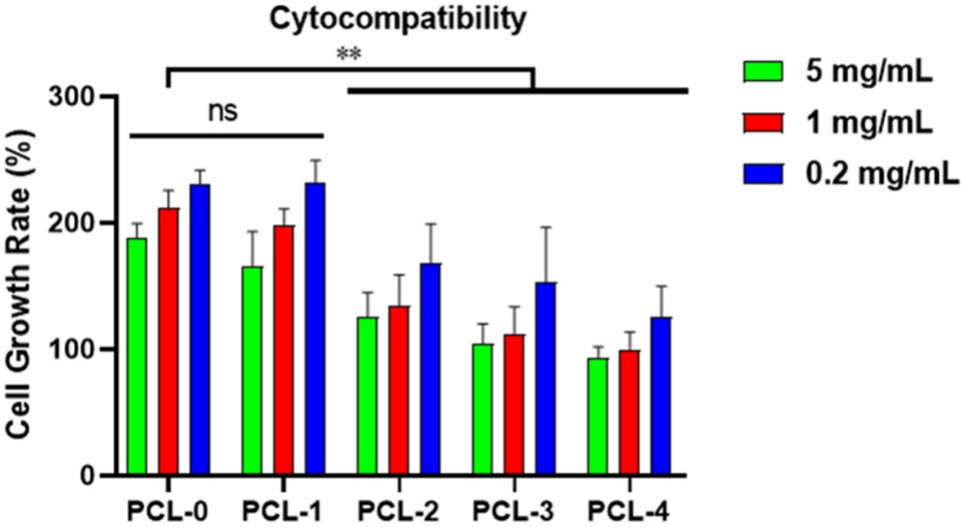
Cytocompatibility of PCL-based membranes. (**p < 0.01).

The cytotoxicity testing was performed by MTT assays so as to evaluate the biocompatibility of the as-prepared PCL-based nanofiber membrances. It is usually to measure the cell density after the cells have been exposed to the nanofibers’ leaching liquor for 48 h to confirm the cytocompatibility of the material. In Fig. 9, it is noted that all groups show no significant cytotoxicity at different concentrations. The cell viability of all test groups modestly declines as the concentration increases. The group with the lowest survival rate remains close to 100% at the highest concentration (5 mg/mL), which suggests that the PCL/Ti_3_C_2_T_X_/baicalin ternary nanofibrous membranes can be utilized as safe wound dressings.

## Conclusions

In this paper, the electrospun membrances based on PCL/Ti_3_C_2_T_X_/baicalin ternary composites were prepared for wound dressing applications. The SEM observation showed that the presence of Ti_3_C_2_T_X_ nanoflakes could decrease in the diameter of the nanofibers due to the increase in the charge density of the electrospinning solution. The PCL nanofiber membrances containing 3 wt% Ti_3_C_2_T_X_ flakes and 5 wt% baicalin had the smallest mean diameter of 210 nm. The thermal stability of the composite nanofibers was improved due to the barrier effect of Ti_3_C_2_T_X_ nanoflakes. The addition of Ti_3_C_2_T_X_ and baicalin could enhance the hydrophilicity, contributing to the release of baicalin from the nanofibrous membranes. Furthermore, the addition of baicalin could endow the PCL/Ti_3_C_2_T_X_/baicalin ternary nanofibrous membranes with good antibacterial properties. The cytocompatibility test confirmed that all of the PCL-based nanofibrous membranes had good compatibility. The antibacterial PCL/Ti_3_C_2_T_X_/baicalin ternary nanofibrous membranes have great potential for wound addressing applications.

## Supplementary Information

Supplementary Information.

## Data Availability

The datasets used and/or analyzed during the current study are available from the corresponding author on reasonable request.
