# Supplementary material for: Electrospun polycaprolactone nanofibrous membranes loaded with baicalin for antibacterial wound dressing
Source: Sci Rep. 2022 Jun 28;12:10900. doi: 10.1038/s41598-022-13141-0 (PMC9240071; doi:10.1038/s41598-022-13141-0)
Supplement: Supplementary file 1 — Supplementary Information. [file 41598_2022_13141_MOESM1_ESM.docx]

**Supporting Information**

Electrospun Polycaprolactone Nanofibrous Membranes Loaded with Baicalin For Antibacterial Wound Dressing

Weiwei Zeng ^1, 2, #^, Nga-man Cheng ^3, #^, Xia Liang ^2^, Haofeng Hu ^4^, Fulin Luo ^4^,

Jia Jin ^4,^ *, Ya-wei Li ^5,^ *

^1^ The Second People's Hospital of Longgang District, Shenzhen, 518112, China.

^2^ Shenzhen Baoan Women’s and Children’s Hospital, Jinan University, Shenzhen 518102, China.

^3^ Accident and Emergency Medicine Academic Unit, The Chinese University of Hong Kong, Hong Kong SAR, China.

^4^ College of Life Sciences and Medicine, Zhejiang Sci-Tech University, Hangzhou 310018, China.

^5^ Lianshui People's Hospital Affiliated to kangda college of Nanjing Medical University, Huaian 223400, China.

# The authors contributed equally.

* Co-responding authors.

E-mails: [lyw_20120220@163.com](mailto:lyw_20120220@163.com) (Y. W. Li); or [aukaukauk@163.com](mailto:aukaukauk@163.com) (J. Jin).

**
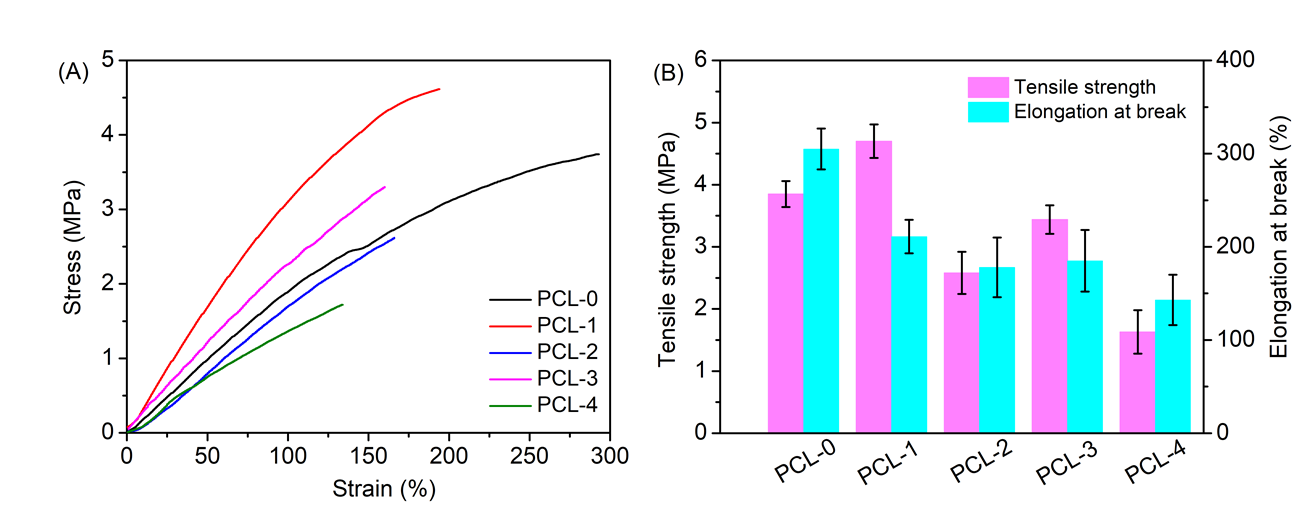
Figure S1.** (A) Stress versus strain curves, (B) tensile properties of PCL composites.

The rectangular tensile specimens were tested at a fixed speed of 1 mm/min on an Instron 5566 universal testing machine. The reported values were the average of at least five successful samples.

**Table S1.** Evaluation standard of inhibition zone according to the standard “SNV 195920-1992” evaluation model.

| Grade | Performance |
| --- | --- |
| Good | presence of inhibition zone >1 mm |
| Fairly good | presence of inhibition zone <1 mm |
| Sufficient | absence of bacterial growth on the sample |
| Limited | limited bacterial growth on the sample |
| Not sufficient | sample is partially or totally rehabilitated by the bacteria |
